# Tourniquets and exsanguinators: a potential source of infection in the orthopedic operating theater?

**DOI:** 10.3109/17453670902930016

**Published:** 2009-04-01

**Authors:** Stephen A Brennan, Raymond J Walls, Elizabeth Smyth, Talal Al Mulla, John M O'Byrne

**Affiliations:** Cappagh National, Orthopaedic HospitalDublinIreland

## Abstract

**Background and purpose** Fomites are increasingly being recognised as a source of hospital-acquired infection. We have therefore assessed tourniquets and exsanguinators for the presence of bacterial pathogens in 1 elective and 2 trauma orthopedic hospitals.

**Material and methods** Swabs were taken prior to and after decontaminating these devices with 1 of 3 different cleaning modalities. These were then assessed for colony counts and organisms identified.

**Results** Bacteria commonly implicated in surgical site infections such as coagulase-negative staphylococci, *Staphylococcus aureus* and *Proteus spp.* were prevalent. We also found a resistant strain of *Acinetobacter* and *Candida.* Exsanguinators were the most heavily contaminated devices, and colony counts in the trauma hospitals were up to 400% higher than in the elective hospital. Alcohol- and non-alcohol-based sterile wipes were both highly effective in decontaminating the devices.

**Interpretation** Infectious organisms reside on the tourniquets and exsanguinators presently used in the orthopedic theater. These fomites may possibly be a source of surgical site infection. We have demonstrated a simple and effective means of decontaminating these devices between cases.

## Introduction

The combined use of a tourniquet and exsanguinator is common practice in orthopedic surgery. Infection following total knee arthroplasty is an infrequent but serious complication, with a reported incidence of 1–2% (Leone and Hanssen 2005). Deep infection complicating knee arthroscopy and anterior cruciate ligament reconstruction is also infrequent, with rates of 1% and 2%, respectively (Armstrong et al 1992, Schollin-Borg et al 2003). Known risk factors associated with deep infection in these procedures include rheumatoid arthritis, intraarticular and oral steroids, ulcers of the skin, previous surgery, recurrent urinary tract infections, and prolonged operating times (Wilson et al. 1990, Papavasiliou et al. 2006).

Highly infectious pathogens including methicillin-resistant *Staphylococcus aureus* (MRSA) have not only been cultured from the skin and nasopharynx of healthcare workers and patients but also from many surfaces of the healthcare environment—including pens, keyboards, stethoscopes, doctors’ white coats, privacy curtains, venepuncture tourniquets, and blood pressure cuffs (Banerjee et al. 1999, Bures et al. 2000, Loh et al. 2000, Rourke et al. 2001, Das et al. 2002, Guinto et al. 2002, Walker et al. 2006, Kaminski et al. 2007).

There is, however, a relative lack of data on the use of limb tourniquets and exsanguinators in the operating theater and their possible role in surgical site infection (SSI). In addition to this lack of data regarding the colonization rates and type of colonization, there is also an absence of guidelines on proper and effective maintenance of these devices. With this in mind, our objective was to examine these devices for colonization by microorganisms in order to determine the extent to which they may play a role in surgical site infection. Having ascertained that they can be contaminated, we examined the efficacies of different sterilization methods.

## Material and methods

### Determination of type and extent of infection

Three dedicated orthopedic centers participated in this multicenter study. 1 elective orthopedic unit (A) and 2 university-affiliated orthopedic trauma units (B and C) were assessed. All sampling took place at the end of the working day, and after the cleaning staff had finished decontaminating the theaters. Swabs were taken from 5 tourniquets and 2 exsanguinators in each of the hospitals. Face masks, sterile gloves, and gowns were worn throughout sampling in order to prevent cross-contamination of the devices under investigation. 2 swabs were from the inner surface and 2 were from the outer surface of each device. All samples were taken by the same investigator.

The swabs (Copan, Murrieta, CA) were then used to streak Columbia chocolate agar plates, which were then incubated aerobically at 37°C for 48 h. The number of colony forming units was recorded. Gram stain, catalase test, and a biochemical identification system (API 20E) were used to identify 20 separate colonies, based on macroscopic morphology.

### Effectiveness of sterilization techniques

Approximately 1 min was spent decontaminating the devices (tourniquets and exsanguinators) by 1 of 3 methods: hospital A (elective): alcohol-free wipes (Fresh’n’Wipes; Vernon Carus, Lancashire, UK); hospital B (trauma): 70% isopropyl alcohol-based wipe (Spectrum; Johnson and Johnson, Skipton, UK); hospital C (trauma): soap (Hydrex Surgical Scrub; Adams, Leeds, UK) and tap water with unsterile paper towels.

The devices were then allowed to dry for 15 min. In the case of the exsanguinators, the inner and outer surfaces were exposed to air approximately every 7 min. The devices were then re-swabbed in each of the 4 locations that were swabbed initially, and these swabs sent for microbiological analysis as previously outlined.

### Correlation with infected cases

We analyzed a database of positive cultures isolated from infected cases that had been treated in the elective orthopedic hospital over the previous 3 years (January 2005 to December 2007). Only information from lower-limb cases that had been treated with the aid of a tourniquet and exsanguinator were extracted from the database. The type of organism and the proportion of infections that it accounted for were recorded.

### Statistics

Mean numbers of colony forming units per device were compared between units A, B, and C using unpaired t-tests. The proportion of swabs positive for bacterial growth was compared between hospitals using a chi-squared test or Fisher’s exact test.

## Results

### Type and extent of infection

The overall mean number of colony forming units (CFUs) per swab prior to cleaning of the devices was lowest in the elective orthopedic hospital (A) at 5 (0–41). In trauma hospitals B and C mean number of CFUs was 15 (0–100) and 21 (0–100), respectively. The level of contamination of tourniquets was highest in hospital B at 9 CFUs (0–74) as compared to 2 (0–13) and 3 (0–28) in hospitals A and C, respectively. The mean number of CFUs per swab prior to cleaning of the exsanguinators in hospital A was 13 (0–41). In hospitals B and C, this value was 31 (0–100) and 65 (0–100), respectively (Table 1).

**Table 1.** Results of culture from swabs from tourniquets and exsanguinators with mean number of colony forming units (CFUs) before and after decontamination

	Swabs positive for bacterial growth	CFUs/swab before	CFUs/swab after
*Combined results*			
Hospital A	14/28	5.3	0
Hospital B	17/28	15	0.04
Hospital C	18/28	21	0.5
*Tourniquets*			
Hospital A	10/20	2.3	0
Hospital B	12/20	8.8	0.05
Hospital C	11/20	2.8	0.3
*Exsanguinators*			
Hospital A	4/8	13	0
Hospital B	5/8	31	0
Hospital C	7/8	65	1.1

Of the organisms identified, 8 were *Staphylococcus epidermidis*, 2 were *Staphylococcus capitis*, 2 were *Staphylococcus cohnii* and 1 was *Staphylococcus kocuria.* 3 colonies of methicillin-sensitive *Staphyloccus aureus* were also identified in addition to 2 of *Proteus mirabilus* and 1 each of *Candida* and beta-lactamase producing *Acinetobacter* (Table 2).

**Table 2.** Comparison of organisms isolated from infected lower limb cases treated in the elective orthopedic hospital with the organisms isolated from exsanguinators and tourniquets. Numbers of colonies identified are given in parentheses

Database	Exsanguinators and tourniquets
Coagulase negative staphylococci 39%	Coagulase-negative staphylococci
Methicillin-sensitive *Staphylococcus aureus* 17%	*Staphylococcus epidermidis* (8)
Methicillin-resistant *Staphylococcus aureus* 12%	*Staphylococcus capitis* (2)
*Proteus mirabilis* 8.5%	*Staphylococcus cohnii* (2)
*Pseudomonas spp.* 7%	*Staphylococcus kocuria* (1) **^a^**
*Streptococcus spp.* 4%	Methicillin-sensitive *Staphylococcus aureus* (3)
*Enterococcus spp.* 2%	*Proteus mirabilis* (2)
*Candida spp.* < 1%	*Candida albicans* (1) **^a^**
	*Acinetobacter* (1) **^a^**

**^a^** These organisms were isolated only from exsanguinators.

### Effectiveness of sterilization techniques

No colony forming units were detected in hospital A following decontamination with the non-alcohol based wipes. In hospital B, only 1 swab (1/28) produced a positive culture following decontamination of devices with alcohol-based wipes. 8 of 28 swabs were positive following decontamination with soap and water in hospital C. The mean number of CFUs per swab after decontamination in hospital C was 1 (0–6) (Table 1).

### Correlation with infected cases

Coagulase-negative staphylococci were the most common organism isolated from infected lower limb cases (Table 2).

## Discussion

Orthopedic surgical site infection prolongs hospital stay by a median of 2 weeks per patient, doubles re-hospitalization rates, and can increase healthcare costs by 300% (Whitehouse et al. 2002). Risk factors for surgical site infection may be divided into those related to the patient, to the type of operation, or to the environment. The main factors in orthopedic patients include age, American Society of Anaesthesiologists (ASA) grade, co-morbidities, obesity, additional nosocomial infections, long preoperative stay, and corticosteroid therapy (de Boer et al. 1999, 2001, Evaillard et al. 2001, Ercole and Chianca 2002). Prevalence studies have shown that the SSI rate is higher in patients with a history of trauma, emergency surgery, and contaminated wounds (Sohn et al. 2002).

Our results show a substantial difference in the mean number of colony forming units per swab when comparing the elective orthopedic hospital with both trauma hospitals. This is not surprising, given the higher throughput and the greater number of infected and open cases in the trauma setting. However, it may reflect better cleaning practices in the elective unit and does raise the possibility that high bacterial loads on these devices may contribute to higher infection rates in acute hospitals.

The tourniquets in use in hospital B showed evidence of wear. It was not common practice for the tourniquets to be cleaned between cases in hospital B, and this was reflected in the results with a higher number of CFUs per swab than in hospitals A and C.

The manufacturers of the Rhys-Davies exsanguinator recommend that it should be suitably clean before being reused, and suggest washing with soap and water. They also advise placing the device in its blue polyethylene sleeve that is supplied with it, and storing it in its cardboard box (Table 3). Regular cleaning of the exsanguinators took place in the elective hospital, but none of the hospitals surveyed followed these storage recommendations. Subsequently, the average number of colony forming units in the elective hospital was lower than in each of the acute hospitals.

**Table 3.** Manufacturer’s recommendations for maintenance and storage of the Rhys-Davies exsanguinator

Ensure that the product is clean and suitable before next application
Pressure of the exsanguinator should be equivalent to 60 mmHg
The exsanguinator may be washed using soap and water
It should not come into contact with solvents
Visually inspect device for signs of cracking or splitting, and if so refer to supplier
The recommended period of use of the device is 12 months
It should be stored in its blue polyethylene bag and placed in its cardboard box
Storage temperature 15 ± 10°C
It should be stored away from electrical equipment

The isolation of high numbers of pathogenic organisms that are associated with surgical site infection from the surfaces of these devices highlights the possibility of transmitting such organisms to patients’ limbs immediately before surgery, thus increasing the risk of postoperative surgical site infection. A study by Ballal et al. showed that when the Rhys-Davies exsanguinator was used sequentially, and without mechanical decontamination with soap and water between patients, that swabs taken consecutively gave an increasing number of CFUs. This was particularly true of swabs taken from the inner surface of the exsanguinator (Ballal et al. 2007).

The most common organisms that cause deep wound infection are *Staphylococcus aureus* and coagulase-negative staphylococci such as *Staphylococcus epidermidis* (Periti et al. 1998, Mauerhan et al. 1994). Although not confirming a causal relationship between colonization on theater equipment and surgical site infection, it was interesting to note the high prevalence of *Staphylococcus* and *Proteus*, both on these devices and in the database of infected cases (see Table 2). There was no MRSA isolated in our study; however, we did isolate a resistant strain of *Acinetobacter.* This is a Gram-negative coccobacillus that is normally a commensal organism but it can also be a nosocomial pathogen. It has previously been implicated in an outbreak of pneumonia in ventilated patients in the intensive care setting, and more recently it was the predominant organism to be isolated in the early stages of osteomyelitis in military personnel (Husni et al. 1999, Yun et al. 2008).

By the very nature of our study, there is always the possibility of cross-contamination with one’s own pathogens at the time of sampling. This is a potential weakness of the study. We tried to ensure that cross-contamination was kept to a minimum by strict adherence to the sampling protocols outlined above. In addition to this we feel that, given the consistently low levels of bacterial pathogens found after decontamination, cross-contamination was almost certainly negligible.

All methods of cleaning significantly reduced contamination from baseline. We note that in hospital C, the level of initial contamination was highest. Decontamination with soap and water did have a statistically significant effect on the levels of colony forming units present; however, this was the only modality that failed to completely eradicate the microorganisms. The operating theater is a complex ecosystem, and many interventions are necessary for optimal infection control. Interventions commonly employed include the use of antibiotic prophylaxis, preoperative skin antisepsis, surgical scrub, gowning, occlusive drapes, aseptic technique, irrigation of the wound, laminar air flow, ultraviolet lighting, air exhaust systems, and closing of operating theater doors (Fletcher et al. 2007). In spite of this, routine decontamination of tourniquets and exsanguinators between cases is not common practice in many hospitals. Given their proximity to the wound and direct application of exsanguinators to the skin before surgery, we feel that this should be the case.

One may argue that the exclusion of tourniquets from the operative field by surgical draping precludes them from being implicated in surgical site infection. However, the passage of bacteria through fabrics used in the operating theater has been identified as a source of wound contamination (Laufman et al. 1979). Previous studies have confirmed that organisms implicated in surgical site infection such as *Staphylococcus epidermidis* and *Streptococcus sanguis* strike through dry polyester/cotton drapes within 30 min, and that this is enhanced when the drapes are soaked with normal saline or blood (Blom et al. 2002). Although disposable non-woven drapes have been shown to be superior in resisting bacterial penetration, a study examining 6 brands of disposable drapes showed that none were impenetrable to bacteria after 90 min (Blom et al. 2000, 2007).

In conclusion, due to the nature of their constituent material, tourniquets and exsanguinators cannot readily be sterilized. This has led to poor maintenance, and to their colonization with organisms that have been implicated in surgical site infection. Implication of a contaminated fomite as the sole vector of cross-infection may be impossible, but an attempt to reduce the bioburden in the immediate vicinity of the patient is certainly advisable.
